# Lymphoplasmacyte-rich meningioma with hematologic signs and PD-L1 over-expression

**DOI:** 10.4322/acr.2021.394

**Published:** 2022-08-12

**Authors:** Gabriele Gaggero, Michela Campora, Davide Taietti, Giannamaria Cerruti, Enrico Lo Bue, Monica Truffelli, Marco Ceraudo, Pietro Fiaschi

**Affiliations:** 1 IRCCS Ospedale Policlinico San Martino, Pathology Unit, Genoa, Italy; 2 Public Healthcare Trust of the Autonomous Province of Trento, Santa Chiara Hospital, Department of Laboratory Medicine, Pathology Unit, Trento, Italy; 3 Azienda Socio Sanitaria Territoriale (ASST) Ospedale Maggiore, Anatomic Pathology Unit, Crema, Italy; 4 IRCCS Ospedale Policlinico San Martino, Molecular Diagnostic Unit, Genoa, Italy; 5 IRCCS Ospedale Policlinico San Martino, Division of Neurosurgery, Genoa, Italy; 6 University of Genoa, Department of Neurosciences, Rehabilitation, Ophthalmology, Genetics, Maternal and Child Health (DINOGMI), Genoa, Italy

**Keywords:** Meningioma, PD-L1, Microenviroment

## Abstract

Lymphoplasmacyte-rich meningioma (LPRM) is one of the rarest variants of grade I meningiomas. It can be clinically associated with prominent peripheral blood abnormalities, anemia, and/or various gammopathy, which usually disappear after surgical removal of the tumor. We document a case of right frontal LPRM in a 72-year-old male who presented general cognitive decadence. The patient suffered from mild anemia. The LPRM is a rare variant of meningioma, with only a few cases globally reported in the literature. It has been categorized as a grade I tumor in the 2021 World Health Organization (WHO) classification central nervous system. Due to the rarity, this meningioma variant origin and biological behavior are still not clear. Immunohistochemistry profile showed prominent PD-L1 expression, leading to additional interrogation on LPRM immunomorphological characteristics, the significance of the inflammatory tumoral microenvironment and its correlation with the immune-checkpoints.

## INTRODUCTION

Meningiomas are the most common non-glial intracranial tumors, accounting for about 39% of all primary brain lesions.^1^ They originate from the arachnoid cap cells of the spinal cord and the brain meningeal coverings. According to the CNS tumors 2021 WHO classification, meningiomas are divided into 3 grades. About 80% of all meningiomas are categorized as grade I,^2^ defined by slow-growing behavior and lengthy survival expectation. The remaining 20% are considered atypical or malignant meningiomas.^3^

LPRM is one of the rarest variants of grade I meningiomas. It can be clinically associated with prominent peripheral blood abnormalities such as anemia and/or various gammopathy, which usually disappear after surgical exeresis.

Generally, the radiologic workup does not help discriminate LPRM from the other variants. The extensive inflammatory cell infiltration may mimic invasion of the adjacent brain tissue,^4^ thus suggesting a malignant behavior at a preoperative stage. Peritumoral brain edema is another frequent feature, probably related to the significant intralesional infiltration and its marked blood supply. In addition, a heterogeneous aspect with cystic components may also be encountered.

Microscopically, LPRM is characterized by conspicuous infiltration of plasma cells, lymphocytes, macrophages, and a variable proportion of meningothelial neoplastic elements, which may be difficult to be detected due to its scarcity. Therefore, the definition of this entity as a neoplastic lesion remains controversial.

The largest review in literature belongs to Firdaus et al.^5^, comprising 164 patients with LPRM retrieved from publications by Zhu et al. (1971-2012),^6^ Yongjun et al. (2002-2013),^7^ Lal et al. (2014)^8^ Tao et al. (2009-2016),^9^ and 21 cases other cases previously reported. Additional 3 cases were described after the article was published.^10^^-^ 12

Most of the described cases are benign, with only a few case reports of cerebral invasion and only one with bone aggression.^10^^,^ 13 ^,^ 14 Despite some hypothesis upon the lesion’s pathophysiology,^6^ mechanisms underlying the meningioma formation and its massive infiltration by lymphocytes, plasma cells and macrophages remains unclear. With the aim of better understanding this unusual entity and its intrinsic pathomechanism, we present our case report, focusing on the characterization of the intratumoral infiltrate and PD-L1 expression by tumor cells.

## CASE REPORT

**Figure 1:**
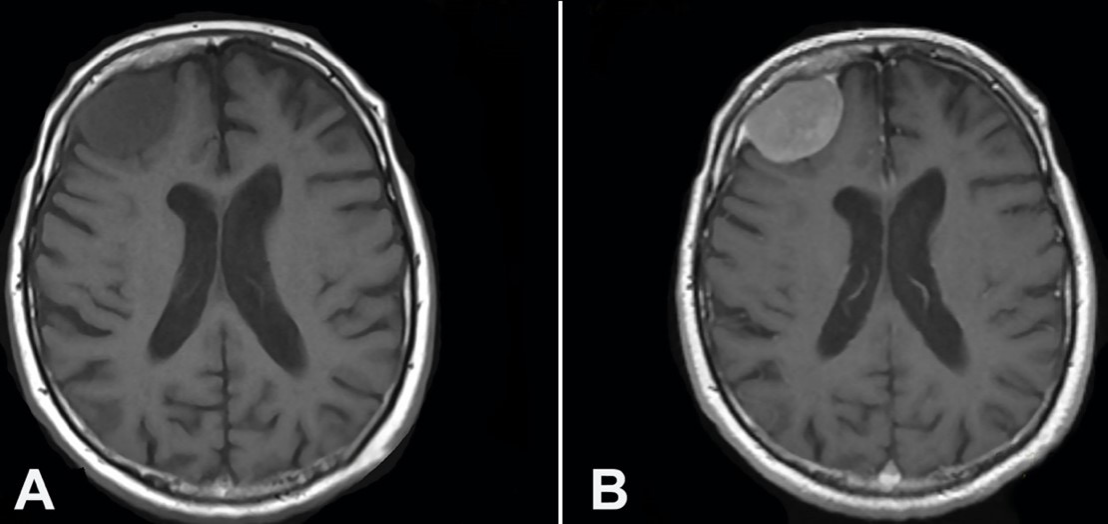
**A–** Preoperative Axial Brain MRI T1 weighted images showing a frontal hypointense extra-axial right lesion; **B –** Preoperative Axial Brain MRI T1 weighted with contrast enhancement showing homogeneous enhancement after gadolinium administration of the frontal right lesion with the characteristic dural tail.

A 72-years-old patient complained of an episode of amnesia and time-space disorientation, presenting a global cognitive impairment over the last months. He was diagnosed with chronic depression with apathy and anhedonia. In the last months, he referred episodes of amnesia and time-space disorientation, presenting a global cognitive impairment. On examination, no focal neurological deficits were present. Brain magnetic resonance imaging (MRI) t revealed a large extra-axial mass (39x27x38 mm) in the right frontal lobe with surrounding periosteal reaction. A substantial amount of the underlying cortical surface was preserved with minimal and flattening of the gyral architecture. No edema, midline shift, or lateral ventricle compression were identified (Figure 1).

The tumor removal extended to the dural margin and frontal bone, leading to Simpson 1 extent of resection.^11^ Macroscopically, the tumor appeared purplish yellow, cauliflower-shaped, soft in consistency, and moderately vascularized with a broad dural base. A defined cleavage plane with the brain parenchyma was present. After the tumor removal, the internal cortical bone adjacent to the lesion was drilled. Then the bone was put back and fixed with plates. A post-surgery CT scan demonstrated a complete removal without acute complications. The patient’s dementia did not improve in the postoperative, and the neurological examination remained unaltered.

## PATHOLOGICAL FINDINGS

**Figure 2:**
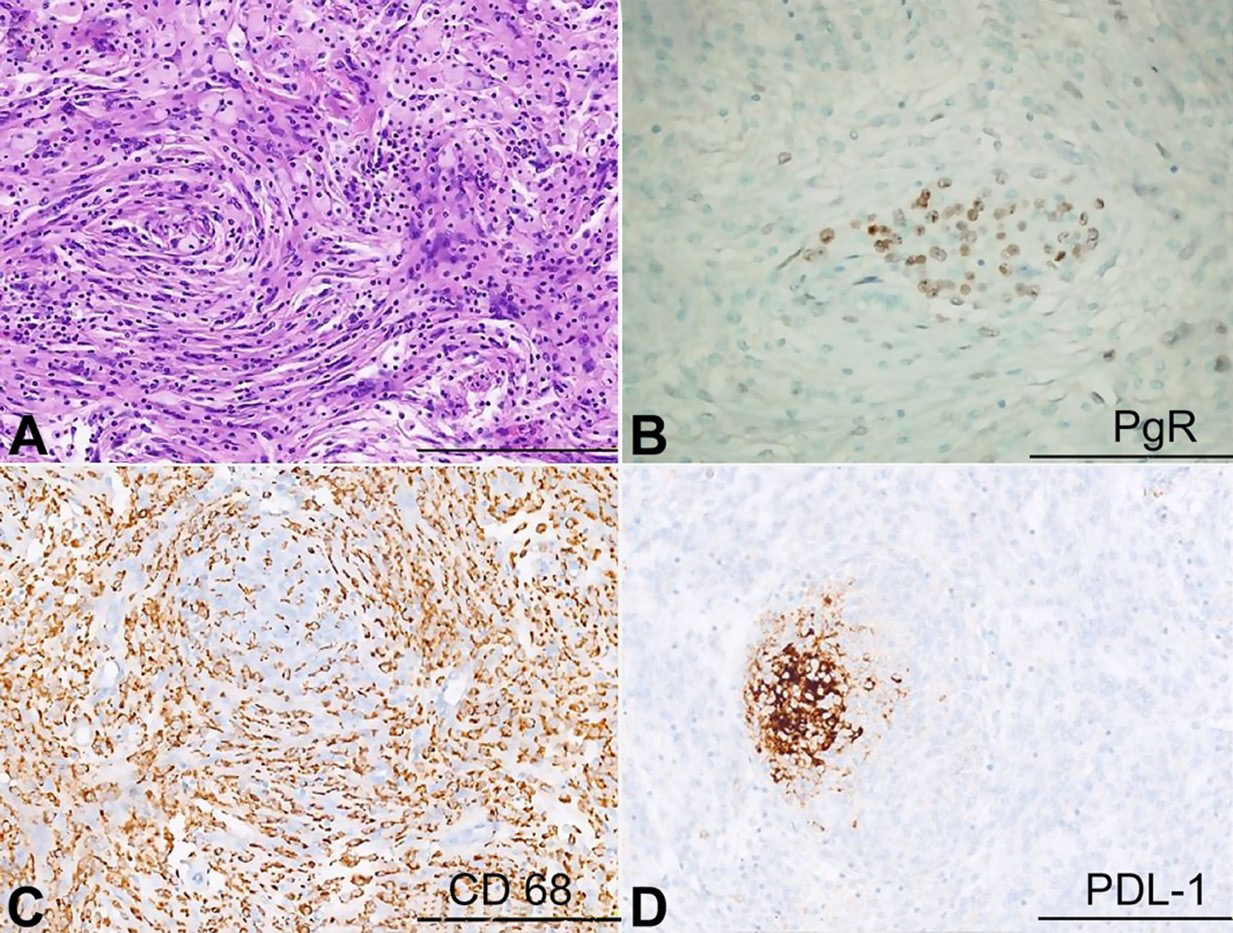
**A–** whorls meningothelial elements with massive infiltration of inflammatory cells (H&E); **B –** Progesterone Receptor (PgR) immunohistochemical staining: an intense nuclear pattern is expressed in meningothelial cells; **C –** CD68/PG-M1 immunohistochemical staining: an intense and diffuse granular pattern is expressed in histiocytes; **D –** PDL-1 immunohistochemical staining: an intense cytoplasmatic and membranous positivity in the meningeal tumoral component. Scale bars: 400 µm (A-C-D), 200 µm (B).

Histopathological examination showed a massive lymphocytic infiltration, dominating and almost obscuring the lesion’s neoplastic epithelial component (Figure 2A). The latter was constituted by small meningeal whorls highlighted with positive immunohistochemistry reactions for epithelial membrane antigen (EMA) (Clone E29; Dako, Glostrup, Denmark) and Progesterone (PgR) (clone 1A6; Dako, Glostrup, Denmark; 1:800) (Figure 2B). Neither areas of hypercellularity, necrosis, increased number of mitoses, nor giant or atypical cells were seen in the meningeal component. Immunohistochemical profiling of the inflammatory infiltrate revealed a strong and predominant CD68 (clone KP1; Thermo Scientific, USA, 1:100) positive population (Figure 2C) and a secondary minority inflammatory component, negative for CD68 and positive for CD3 (clone L22; Biocare Medical, CA, USA, 1:100) and CD20 (clone BC33; Biocare Medical, CA, USA, Prediluted). The Ki-67 (clone MIB1; Dako) antigen/mitotic count evaluated on 1000 cells was 3.6%. The overlying parietal bone was found to be involved in the tumor. A final diagnosis of LPR meningioma WHO grade I was made.

We completed the immunohistochemical profile with PD-L1 evaluation (mouse monoclonal, Clone 22C3, EnVision FLEX in Autostainer Link 48, Dako; 1:50), which substantially retraces EMA and Progesterone positivity, exhibiting an almost exclusive reaction in the meningeal tumoral elements (Figure 2D).

**Figure 3:**
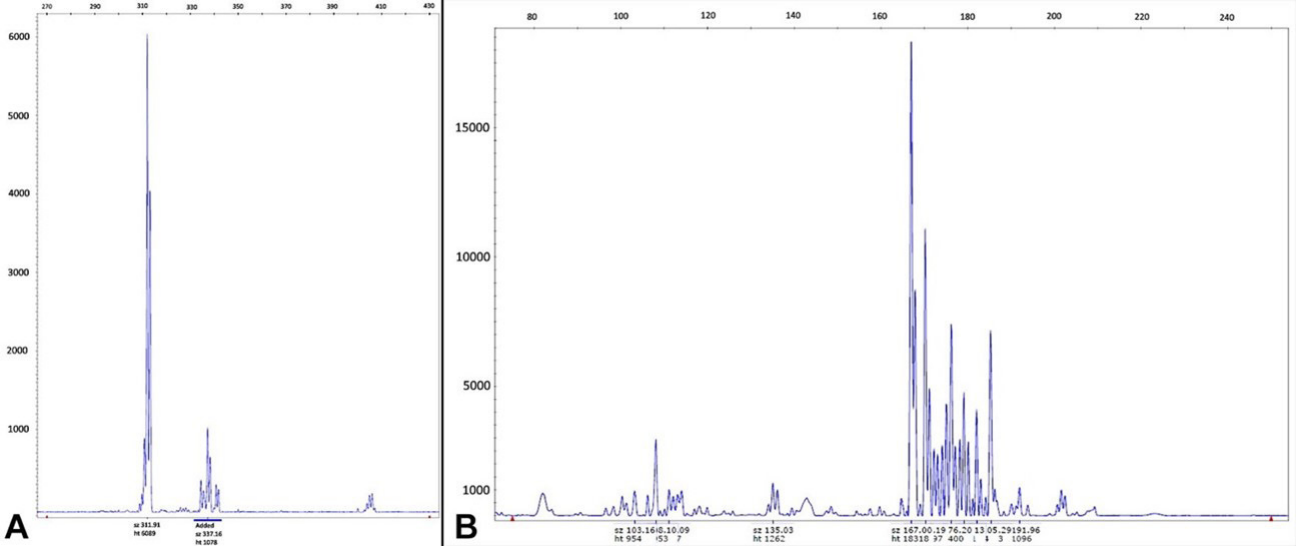
**A–** Clonality assessment of the inflammatory tumoral population of LPR. Results of the gene rearrangement profile showing Fr3-JH peak interpreted as a clonal B cell overexpression in the context of a polyclonal pattern; **B** – PCR performed to analyze the human T-cell rearranging gamma gene (TCRG) locus rearrangements, revealing the presence of an irregular polyclonal T lymphocyte pattern.

The inflammatory component was also elaborated through PCR analysis. Heavy chain immunoglobulin (IgH) gene rearrangements were studied using DNA extracted from FFPE sample, using three complementary amplification primers for the fragments Fr1-JH, Fr2-JH and Fr3-JH. The last fragment (Fr3-JH) resulted over-expressed, creating a Gaussian-like curve together with other peaks, and was interpreted as a clonal B cell overexpression in the context of a polyclonal pattern. Two other different PCR were performed with the same technique to analyze Fr3-J rearrangements of the human T-cell rearranging gamma gene (TCRG) locus, revealing an irregular polyclonal T lymphocyte pattern (Figure 3).

## DISCUSSION

In 1971, Benerjee and Blackwood^15^ first described a sub frontal tumor showing combined plasmacytoma and meningioma features. According to the largest review in the literature,^5^ LPRM shows a higher rate in young and middle-aged patients without sex predominance.

After over 40 years since its first description, the peculiar aspect of this meningioma and its massive infiltration of lymphocytes, plasma cells, and macrophages remain unclear. Weidenheimet et al.^16^ considered the lymphoplasmacytic infiltrations an immune response to the tumor antigenicity, while Gregorios et al.^17^ hypothesized that these cells differentiated from a totipotent cluster of mesenchymal cells, like a chronic and continuous inflammation surrounding the neoplastic meningothelial component.^17^

Systemic hematological anomalies such as hypergammaglobulinemia and refractory iron anemia have been documented in some patients.^18^ However, the relationship between inflammatory lesions, systemic hematological abnormalities, and this meningioma variant remains uncertain. This particular phenomenon had only been reported in 2 subtypes of meningiomas: LPRM (WHO grade I) and chordoid meningioma (WHO grade II).^19^ The significance of hematological abnormalities, in this type of meningioma is still unclear, requiring more cases and long-term follow-ups to answer this question.

Gi et al.^20^ reported a 14-year-old boy with a recurrent meningioma and a high serum immunoglobulin level that immediately decreased after surgery and increased again with tumor recurrence.^20^

Our patient presented mild anemia, and protein electrophoresis did not show M band or hypergammaglobulinemia. However, proteins like albumin and gamma-globulins were within the normal range.

Kepes et al.^21^ reported that chordoid meningioma is more likely to be accompanied with LPRM and causes Castleman’s syndrome in children and young adults (delayed somatic and sexual development, hepatosplenomegaly, iron refractory microcytic hypochromic anemia, and bone marrow plasmacytosis with dysgammaglobulinemia).^21^

Thus, we speculate that peculiar histological manifestations and the high proportion of dysgammaglobulinemia and/or iron refractory hypochromic microcystic anemia in LPRM patients may imply similar pathogenesis of these two variants.

According to Stam,^22^ the abundant production of almost all classes of immunoglobulins suggests a non-tumoral plasma cell infiltrates.

The expression of immune checkpoints within the solid tumor microenvironment is an important factor in tumor-induced immunosuppression and evasion of the innate immune response to malignancy. Like many other tumors, meningiomas can upregulate the expression of PD-L1.^23^^-^ 26

This is the first report that focuses on PD-L1 expression in LPRM: similar papers were not found in the published literature; no results are reported after a search in PubMed database using “lymphoplasmacyte-rich meningioma” or “LPRM” and “PD-L1” as keywords. LPRM is commonly considered a benign lesion, the increased PD-L1 expression is an interesting matter of speculation. According to the literature, PD-L1 expression in meningiomas should be connected to a more aggressive propensity. Some studies have postulated that a significant correlation between PD-L1 expression and meningioma grade is present.^24^^,^ 27 ^,^ 28 LPRM is generally described as a subtype characterized by benign behavior. The role of immune checkpoints expression in solid tumors’ microenvironment is currently recognized as a major factor in tumor-induced immunosuppression and escape of the cancer- innate immune response. PD-L1 expression, and regulatory T-reg cells, are supposed to contribute to the immunosuppressive tumor microenvironment. In particular, meningiomas, among solid tumors, can also express and upregulate PD-L1 expression.

Differently from other meningioma subtypes, the increased expression of PD-L1 seems unrelated to meningioma grading. However, it could be explained by the tendency of LPRM to interact with the microenvironment peculiarly. The increased PD-L1 expression may be correlated to the different stromal populations concerning other meningiomas subtypes.

Several mechanisms can potentially induce PD-L1 expression, including hypoxia, activation of oncogenic and inflammatory signaling pathways, cytokine release, and epigenetic regulatory mechanisms. PD-L1 expression has not only been used to predict response to immune checkpoint therapy but also shows prognostic significance for tumor progression in several cancers.^27^^,^ 29

Han et al.^24^ and Du et al.^26^ demonstrated a worse prognosis associated with increased PD-L1 expression in the meningiomas, with intratumoral PD-L1 expression highest in grade III.

Han^24^ postulated that the degree of PD-L1 expression is predictive of poor survival only in grade II and III cancers, requiring a large number of samples to identify the difference. Studying the systemic and local immunosuppression in patients with high-grade meningiomas, Li et al.^28^ was unable to identify such an effect, given the limited number of high-grade tumors in his cohort.

As reported in other tumors such as HCC or pancreatic ductal adenocarcinoma, the overexpression of PD-L1 may be mediated by an inflammatory microenvironment involving macrophages. PD-L1 expression is positively related to macrophage infiltration within the tumor stroma. Tumor-infiltrating macrophages could produce TNF-α to increase PD-L1 expression on tumor cells and stabilize PD-L1 via the NF-κB pathway.^30^ The conspicuous macrophage infiltration in LPRM may induce PD-L1 overexpression of meningioma cells, as demonstrated in our case unless a clear influence on tumor aggressiveness.

The intratumoral inflammatory component was also analyzed through a PCR. In line with current theories regarding the tumor pathobiology, the hypothesis was that plasma cell infiltration was related to blood abnormalities and that the intralesional inflammatory cell reaction should be considered secondary, presumably reflecting an additional unusual immunological host’s response.^6^

Immunological constituents play an important role in the tumor microenvironment, and the surrounding cellular population. Many studies focused on filtering lymphocytes, natural killer (NK) cells, macrophages, dendritic cells, eosinophils, mast cells, and immature myeloid cells in human cancers.^31^^-^ 41

Our results suggested the presence of both a clonal B cell overexpression in the context of a polyclonal pattern and an irregular polyclonal T lymphocyte population. We can assume that a complex and continuous cross-reaction among different inflammatory actors, comprehensive of B and T lymphocytes and macrophages, contributes to creating the tumoral microenvironment. On the other hand, neoplastic cells elicit an immune response, probably creating clonal and atypical populations, as shown in our PCR spectrum.

The role of tumor-infiltrating B cells was studied in ovarian, lung, breast, and cervical cancers: at the tumor site, B lymphocytes can function as antigen-presenting cells to facilitate the persistence of CD8+ T lymphocytes for long periods, improve T lymphocyte responses by producing antibodies, produce cytokines to promote the organization of local lymphoid structure and change the relationship between Th1, Th2 cells.^31^^,^ 42 ^,^ 43

Han et al.^24^, studying the expression and prognostic impact of immune modulatory molecule PD-L1 in meningiomas, noticed CD20+ cell infiltration predicted a favorable progression-free survival.

Furthermore, like in primary melanoma, a brisk infiltration of T cells is a favorable prognostic factor; similar data have been found in other cancers, including ovarian, renal cell carcinoma (RCC), bladder, and several other solid cancers.^41^^,^ 44 ^-^ 46

T regulatory cells (Tregs) can suppress proliferation, produce cytokines and induce the cytolytic activity of CD4+ and CD8+ T cells by mechanisms involving cell-to-cell contact and the release of cytokines such as TGF-β. Tregs can also induce an immunosuppressive phenotype in other cell types such as macrophages.^31^^,^ 47

More studies are necessary to understand this complicated mosaic of interaction better.

## CONCLUSIONS

In conclusion, after 50 years of observing this peculiar meningioma, the mechanisms underlying its formation and the significance of the massive infiltration of lymphocytes, plasma cells, and macrophages that characterize it still remain unclear.

With the increasing importance of immunotherapy in solid tumors and the constant progression of therapies in hemolymphatic diseases, we think it is almost mandatory to study the relationship between inflammation and tumoral lesions, possibly leading to an optimized target drug for patients affected with this rare variety of meningioma before surgery, improving patient’s outcome.
